# The relationship between serum monoterpene levels and bone health: a retrospective cross-sectional analysis from the National Health and Nutrition Examination Survey (NHANES) data

**DOI:** 10.3389/fpubh.2024.1436415

**Published:** 2024-08-07

**Authors:** Yu-Wei Fang, Chi-Kang Wang, Chien-Yu Lin

**Affiliations:** ^1^Division of Nephrology, Department of Internal Medicine, Shin-Kong Wu Ho-Su Memorial Hospital, Taipei, Taiwan; ^2^School of Medicine, College of Medicine, Fu Jen Catholic University, Taipei, Taiwan; ^3^Department of Environmental Engineering and Health, Yuanpei University of Medical Technology, Hsinchu, Taiwan; ^4^Department of Internal Medicine, En Chu Kong Hospital, New Taipei City, Taiwan

**Keywords:** bone mineral density, monoterpenes, α-pinene, β-pinene, limonene

## Abstract

**Introduction:**

Monoterpenes, a subset of the terpene family composed of two isoprene units, have garnered significant attention in research circles owing to their potential medicinal benefits. Recent experimental studies indicate that they might exert positive effects on bone health. Nevertheless, the impact of monoterpenes exposure on bone health remains unexplored in humans.

**Methods:**

We examined 748 adults (age ≥ 40 years) from the National Health and Nutrition Examination Survey (NHANES) 2013–2014 to explore the correlation between three monoterpenes (α-pinene, β-pinene, and limonene), bone mineral density (BMD) in the total lumbar spine and proximal femur, FRAX^®^ scores, and prior bone fracture history.

**Results and discussion:**

Our analysis unveiled a significant inverse association between a one-unit increase in the natural logarithm (ln) of α-pinene and limonene and total proximal femur BMD (ß = −0.027, S.E. = 0.008, *P* = 0.004 and ß = −0.019, S.E. = 0.007, *P* = 0.016, respectively). As serum α-pinene levels ascended across quintiles, there was a notable decrease in total proximal femur BMD (*P* for trend = 0.025). The inverse relationship between ln α-pinene levels and total proximal femur BMD was more pronounced in women, especially pre-menopausal women. Compared to subjects with α-pinene and limonene levels at or below the 50^th^ percentiles, those exceeding this threshold exhibited the lowest mean value of total proximal femur BMD (0.8628 g/cm2, S.E. = 0.026, *P* = 0.009). However, the trend was not statistically significant (*P* = 0.070). Additionally, all three monoterpenes were linked to a higher prevalence of previous spine fractures, whereas β-pinene showed a reduced incidence of other types of fractures. In this comprehensive survey of American adults aged 40 and above, higher serum levels of α-pinene and limonene correlated with decreased total proximal femur BMD. Furthermore, our findings suggest a potential combined effect of α-pinene and limonene on total proximal femur BMD. Further investigation is essential to elucidate the clinical relevance and causative nature of our findings.

## 1 Introduction

Monoterpenes, belonging to the terpene family and composed of two isoprene units, are defined by the molecular formula C10H16. These compounds encompass a broad spectrum of naturally volatile metabolites primarily synthesized by plants. They are utilized in essential oils derived from plants such as pine, citrus fruits, and eucalyptus. Monoterpenes are also utilized in gastronomic customs, cleaning products, and beauty formulations, fulfilling a wide range of functions (1). Individuals encounter monoterpenes through respiratory, oral, and dermal exposure. Common sources of exposure include inhaling air in forests, consuming foods and beverages containing monoterpenes, and using household products like air fresheners and cleaning agents. Occupational exposure can also occur in industries such as agriculture, food processing, and manufacturing of personal care products (2, 3). Upon exposure, monoterpenes undergo extensive oxidation reactions, resulting in metabolites that are expelled through urine within a period of 12 to 24 hours (4). Considerable studies have shed light on the therapeutic possibilities of monoterpenes. However, recent studies have connected essential oils that are rich in high concentrations of monoterpenes with potential risks, including neurotoxicity, genotoxicity, or liver toxicity (5, 6). Recent epidemiological research has additionally linked exposure to monoterpenes with cardiovascular risk factors, including elevated fasting glucose levels, hyperlipidemia, and metabolic syndrome (7, 8).

Fractures caused by osteoporosis contribute significantly to disability, placing considerable economic and social strains. Numerous factors influence bone mineral density (BMD), including age, gender, genetics, lifestyle habits, and endocrine disorders (9). Normal bone metabolism involves a delicate balance between osteoblast-mediated bone formation and osteoclast-mediated bone resorption. Osteoblasts, derived from mesenchymal stem cells, are responsible for synthesizing bone extracellular matrix and facilitating mineral deposition, while osteoclasts, derived from hematopoietic stem cells, break down bone tissue to release minerals into the bloodstream (10). Recent research has revealed a link between heightened oxidative stress and a higher risk of fractures (11, 12). Monoterpenes have been studied for their beneficial effects on bone cells. α-pinene, a cyclic monoterpene, has been shown to improved mineralization of osteoblasts and counteract the inhibitory effects on osteoblast differentiation (13). Limonene, a single ring monoterpene, has demonstrated enhancing bone extracellular matrix synthesis, and facilitating mineral deposition in mouse preosteoblasts (14). Additionally, metabolites of monoterpenes have been discovered to suppress osteoclast activity in animal model (15). However, monoterpenes also have properties that may adversely affect bone metabolism. They can generate secondary organic aerosols linked to increased oxidative stress (16–19). This oxidative stress can disrupt the balance between bone formation and resorption, potentially leading to reduced BMD. Moreover, the metabolism of monoterpenes into reactive metabolites may elevate toxicity and oxidative stress (20–22), further impacting bone health by impairing osteoblast function and promoting osteoclast activity (11, 12). These findings underscore the importance of conducting additional research to fully understand the complex impacts of monoterpene exposure on human bone health.

Notably, there have been no prior epidemiological studies investigating the relationship between monoterpene exposure and bone health. To bridge this gap in understanding, we undertook an analysis using data from the National Health and Nutrition Examination Survey (NHANES) 2013-2014. This dataset offered crucial insights into the levels of three serum monoterpenes—α-pinene, β-pinene, and limonene—along with BMD in the proximal femur and lumbar spine, FRAX^®^ scores, and history of previous fractures. Our aim is to undertake comprehensive research to investigate the association between monoterpenes exposure and bone health in U.S. adults.

## 2 Materials and methods

### 2.1 Study population

**Figure 1:**
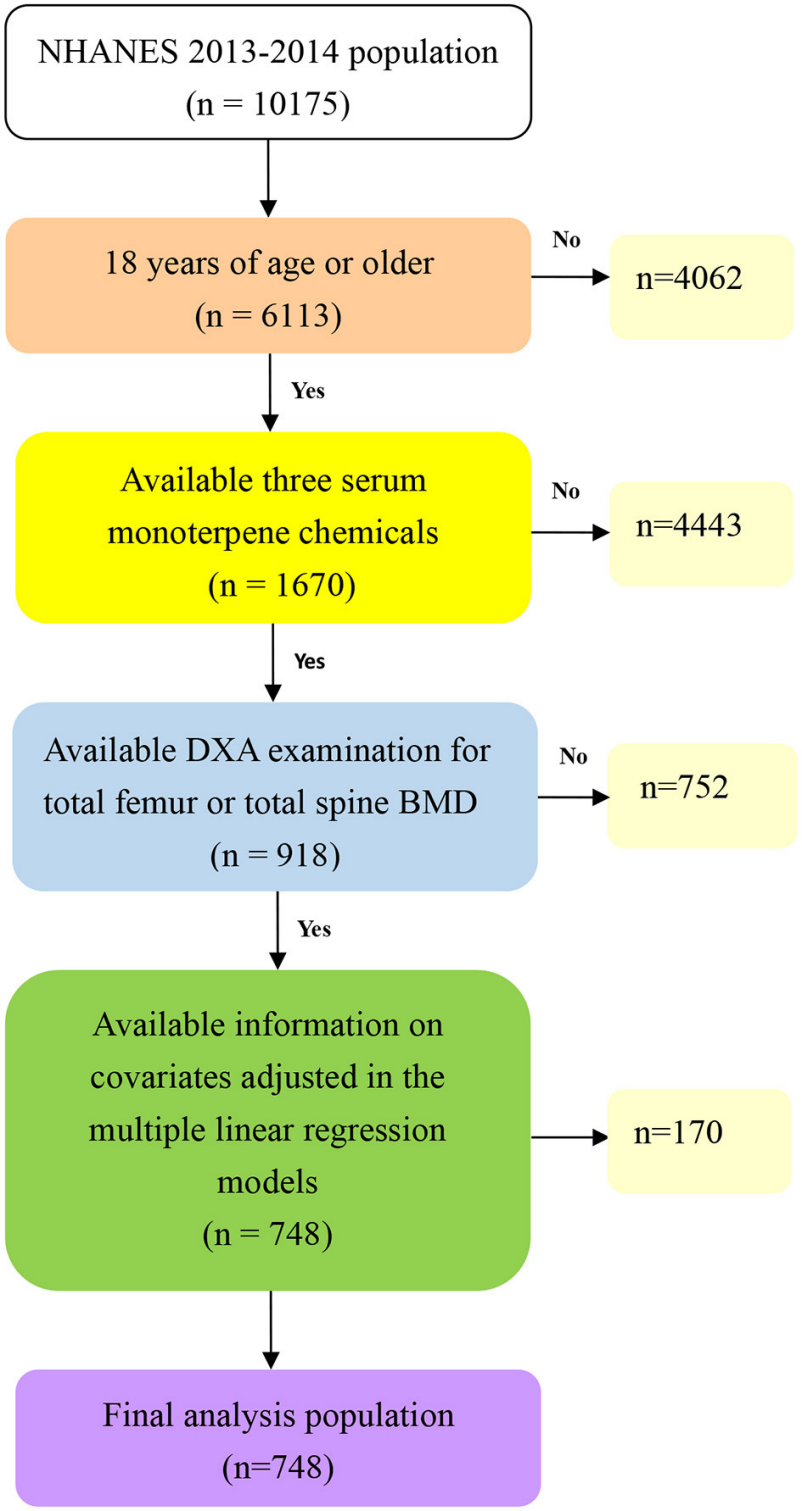
Flow chart algorithm.

NHANES, a biennial national survey, is conducted to offer a comprehensive representation of the U.S. population. Detailed survey methodologies and consent forms can be accessed through the NHANES website (23). For our study, we utilized data from the 2013-2014 NHANES database, comprising 10,175 participants. After excluding those lacking all three monoterpene chemicals, the number of participants was reduced to 2,213. Further exclusions were made for 1,295 participants with incomplete Dual-energy X-ray Absorptiometry (DXA) data. From the initial pool of 918 subjects, an additional 170 individuals were omitted due to incomplete covariate data. Ultimately, our analysis centered on 748 individuals. A visual representation of the selection process is depicted in Figure 1.

### 2.2 Measurement of serum monoterpene levels

Within the NHANES 2013-2014 study, a subset comprising approximately one-third of participants underwent analysis for serum levels of three specific monoterpenes: α-pinene, β-pinene, and limonene. In instances where monoterpene levels fell below the limits of detection (LOD), a value derived by dividing LOD by the square root of 2 was provided. For comprehensive details on the analytical methodology employed in this study, please consult the NHANES website (24).

### 2.3 Measurement of DXA scan

During NHANES 2013-2014, DXA scans were administered, focusing on the proximal femur and lumbar spine among participants aged 40 and older. Exclusions from the examination included pregnancy, recent self-reported use of radiographic contrast material, and self-reported weights exceeding 450 pounds. The femur scans provided bone measurements for various regions including the total proximal femur, femoral neck, trochanter, intertrochanter, and Ward's triangle. Similarly, the spine scans offered bone measurements for the total lumbar spine and vertebrae L1–L4. Skilled technicians utilized Hologic QDR-4500A fan-beam densitometers (Hologic, Inc., Bedford, MA, USA) alongside Apex 3.2 software for conducting the DXA scans. For comprehensive methodological details, interested parties can refer to the NHANES website (25).

### 2.4 FRAX^®^ score

FRAX^®^ scores serve as estimations of the 10-year risk for both hip fracture and major osteoporotic fractures, calculated based on a comprehensive array of fracture risk factors. These factors include age, gender, weight, height, prior fractures, parental history of hip fractures, glucocorticoid usage, rheumatoid arthritis, secondary osteoporosis, current smoking and alcohol consumption, and femur neck BMD. Within NHANES 2013–2014, individuals aged ≥ 40 years with valid data for femoral neck BMD underwent FRAX^®^ score computation. Data on fracture risk factors were derived from NHANES database, and FRAX^®^ scores were calculated using Hologic version 3.05. For this study, the definition of fracture encompasses both self-reported fractures and vertebral fractures identified through DXA scans. Comprehensive information is available on the FRAX^®^ and NHANES websites (26, 27).

### 2.5 Other covariates

In the present study, sociodemographic information, body mass index (BMI), smoking habits, alcohol intake, physical activity quantified by metabolic equivalent scores, menopausal status, serum vitamin D levels, total calcium intake, history of osteoporosis treatment, use of prednisone or cortisone, postmenopausal hormone therapy, and previous fractures (hip, wrist, spine, or other sites) were considered as covariates. Detailed information can be found in the Supplementary material.

### 2.6 Statistical analysis

Statistical analyses were performed utilizing IBM SPSS Statistics (Version 20; SPSS Inc., Chicago, IL), with sampling weights applied as per NHANES website protocols. Complex Sample Survey module of SPSS 20 was utilized for analysis (28). General linear models within the complex samples framework were employed to explore the correlation between monoterpene markers and total lumbar spine/proximal femur BMD across the entire study cohort and various subgroups. To control for covariates, two distinct models were employed. Model 1 adjusted for age, gender, race/ethnicity, BMI, smoking, alcohol consumption, metabolic equivalent scores, serum vitamin D level, and total oral calcium intake. Model 2 included additional variables such as diabetes mellitus, hypertension, history of osteoporosis treatment, use of prednisone or cortisone, and postmenopausal hormone therapy. Statistical significance was determined by consistency across both models (29, 30). Logistic regression analysis of complex samples was used to examine the associations between self-reported fractures and natural logarithm-transformed (ln) serum monoterpene levels, as these covariates exhibited non-normal distributions. To address multiple comparisons, the Bonferroni correction was applied. Given the analysis of three types of monoterpenes, statistical significance in the multiple linear regression analysis was set at *P* < 0.017 (0.05/3).

## 3 Results

The study included 748 participants, with an average age of 58.49 years (SD = 11.80, range 40 to 80 years). Table 1 displays the basic demographic characteristics of the study group. The mean concentrations (SD) of α-pinene, β-pinene, and limonene were 0.10 ng/mL (0.07), 0.010 ng/mL (0.11), and 1.46 ng/mL (1.20), respectively. The detection rates for these three monoterpenes were 76.1%, 75.8%, and 100%, respectively. A correlation existed between the levels of the monoterpenes, where α-pinene and β-pinene displaying the strongest association (Spearman's correlation coefficient, 0.742; *P* < 0.001).

**Table 1:** Baseline characteristics of study participants.

**Variables**	**Mean/numbers**	**SD/%**
Age (year)	58.49	11.80
Female	384	51.30
**Ethnicity**		
Mexican-American	92	12.30
Other Hispanic	58	7.80
Non-Hispanic white	379	50.70
Non-Hispanic black	136	18.20
Non-Hispanic Asian	68	9.10
Other ethnicity	15	2.00
**Smoking status**		
Non-smoker	489	65.40
ETS	117	15.60
Current smoker	142	19.00
Alcohol consumption ≥ 12 drinks/year	557	74.50
**Medical history**		
Diabetes mellitus	160	21.40
Hypertension	378	50.50
BMI (kg/m^2^)	29.18	6.07
**BMD (g/cm** ^2^ **)**		
Total femur	0.95	0.15
Total spine	1.01	0.15
**Monoterpenes (ng/mL)**		
α-pinene	0.10	0.07
β-pinene	0.10	0.11
limonene	1.46	1.20

Tested by Student's 2-tailed t-test or by one-way analysis of variance.

**Figure 2:**
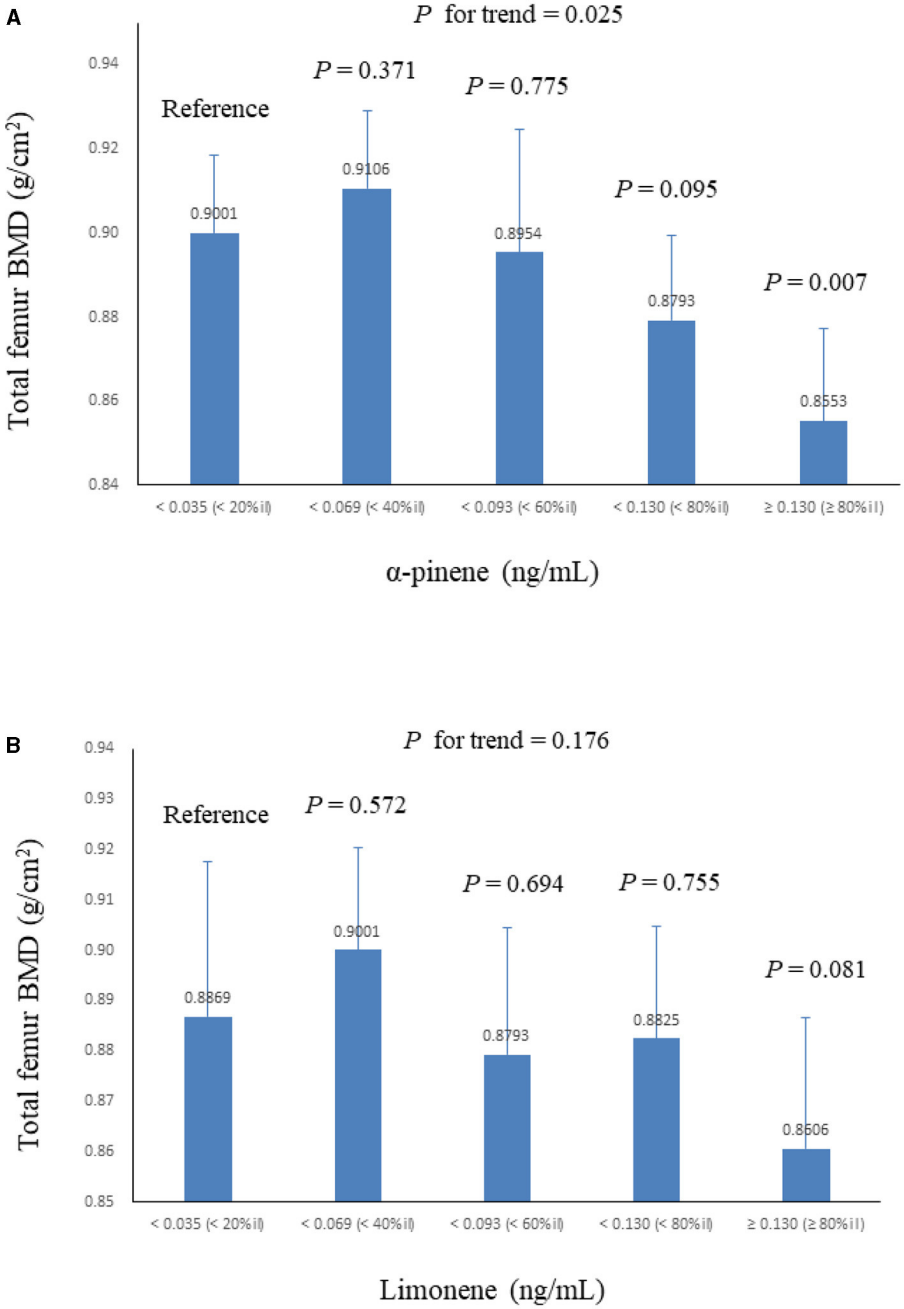
Mean ± S.E. of total femur BMD across quintiles of serum monoterpenes in linear regression models, with results weighted for sampling strategy (adjusted for model 2). **(A)** α-pinene. **(B)** limonene.

Table 2 summarizes the adjusted regression coefficients indicating the differences in total lumbar spine/proximal femur BMD with a one-unit increase in ln monoterpenes. Our analysis revealed a significantly negative association between ln α-pinene and ln limonene and total proximal femur BMD in both models (β coefficient = −0.027, S.E. = 0.008, *P* = 0.004 and β coefficient = −0.019, S.E. = 0.007, *P* = 0.016 in model 2). The correlations between the quintiles of α-pinene and ln limonene and total proximal femur BMD are illustrated in Figure 2. As serum α-pinene levels increased across quintiles, the mean total proximal femur BMD significantly decreased (*P* for trend = 0.025). Moreover, the highest quintile exhibited a significant reduction in total proximal femur BMD compared to the lowest quintile (*P* = 0.007). The mean total proximal femur BMD difference between the upper and lower quintiles of α-pinene was 5.0%. Additionally, the results indicated no statistically significant decrease in total femur BMD with increasing limonene quartiles. Table 3 summarizes the β coefficients reflecting the differences in subregions of proximal femur BMD with respect to a one-unit increase in ln α-pinene and ln limonene in model 2. Our analysis revealed that ln α-pinene was negatively associated with all subregions of proximal femur BMD, while ln limonene was negatively associated with total proximal femur and trochanter BMD.

**Table 2:** Adjusted regression coefficients (S.E.) for differences in total femur and total spine BMD relative to a one-unit increase in natural log-transformed serum monoterpenes, with results weighted for sampling strategy.

	**Total femur BMD (g/cm** ^**2**^ **)**			**Total spine BMD (g/cm** ^**2**^ **)**		
**Serum monoterpenes (ng/mL)**	**Unweighted no./population size**	β **coeff. (S.E.)**	***P value***	**Unweighted no./population size**	β **coeff. (S.E.)**	***P value***
**Ln** α**-pinene**						
Model 1	718/105988465	−0.028 (0.008)	0.004	480/69224111	−0.008 (0.013)	0.541
Model 2	718/105988465	−0.027 (0.008)	0.004	480/69224111	−0.007 (0.013)	0.581
**Ln** β**-pinene**						
Model 1	729/107269168	−0.015 (0.009)	0.133	486/69888259	−0.011 (0.013)	0.400
Model 2	729/107269168	−0.012 (0.009)	0.195	486/69888259	−0.009 (0.013)	0.500
**Ln limonene**						
Model 1	724/106451445	−0.023 (0.008)	0.009	484/69629112	−0.026 (0.010)	0.018
Model 2	724/106451445	−0.019 (0.007)	0.016	484/69629112	−0.023 (0.009)	0.027

Model 1 adjusted for age, gender, race/ethnicity, BMI, smoking, drinking, metabolic equivalent scores, serum vitamin D level, total oral calcium intake.

Model 2 adjusted for model 1 plus diabetes mellitus, hypertension. ever treated for osteoporosis, ever taking prednisone or cortisone, ever using female hormones after menopause.

**Table 3:** Adjusted regression coefficients (S.E.) for differences in subgroups of total femur BMD relative to a one-unit increase in natural log-transformed biomarkers of serum monoterpenes, with results weighted for sampling strategy.

	**Ln** α**-pinene (ng/mL)**			**Ln limonene (ng/mL)**		
**BMD (g/cm** ^2^ **)**	**Unweighted no./ Population size**	β **coeff. (S.E.)**	***P value***	**Unweighted no./ Population size**	β **coeff. (S.E.)**	***P value***
**Total femur**	718/105988465	−0.027 (0.008)	0.004	724/106451445	−0.019 (0.007)	0.016
Femoral neck	718/105988465	−0.026 (0.008)	0.003	724/106451445	−0.015 (0.008)	0.078
Trochanter	718/105988465	−0.021 (0.007)	0.012	724/106451445	−0.019 (0.006)	0.004
Intertrochanter	718/105988465	−0.028 (0.010)	0.009	724/106451445	−0.017 (0.009)	0.067
Wards triangle	718/105988465	−0.032 (0.008)	0.001	724/106451445	−0.016 (0.009)	0.087

Adjusted for model 2.

The interaction between α-pinene and limonene associated with total proximal femur BMD is depicted in Table 4. Using subjects with α-pinene ≤ 50th and limonene ≤ 50th percentiles as a reference, subjects with α-pinene > 50th and limonene > 50th percentiles displayed the lowest mean value of total proximal femur BMD (0.8628 g/cm^2^, S.E. = 0.026, *P* = 0.009). However, the trend was not statistically significant (*P* = 0.07). The associations between total proximal femur BMD and serum α-pinene and limonene in subpopulations are detailed in Table 5. We observed a negative correlation between ln α-pinene level and total proximal femur BMD in the total population (β coefficient = −0.027, S.E. = 0.008, *P* = 0.004), in women (β coefficient = −0.035, S.E. = 0.009, *P* = 0.001), and particularly in pre-menopausal women (β coefficient = −0.040, S.E. = 0.016, *P* = 0.025). Regarding the correlation between limonene and total proximal femur BMD, we found ln limonene level was correlated with a decrease in total proximal femur BMD only in the total population, but not in all subpopulations.

**Table 4:** Mean (S.E.) of total femur BMD in different α-pinene and limonene subgroups in complex sample of multiple linear regression model, with results weighted for sampling strategy.

		**Total femur BMD (g/cm** ^**2**^ **)**			
		**Mean**	**S.E**.	***P*** **value**	***P*** **for trend**
α-pinene ≤ 50%ile	limonene ≤ 50%ile	0.8962	0.02014	Reference	0.070
	limonene > 50%ile	0.8965	0.01817	0.983	
α-pinene > 50%ile	limonene ≤ 50%ile	0.8864	0.02207	0.426	
	limonene > 50%ile	0.8628	0.02552	0.009	

Adjusted for model 2.

**Table 5:** Adjusted regression coefficients (S.E.) for differences in total femur BMD relative to a one-unit increase in natural log-transformed biomarkers of serum monoterpenes in subpopulations, with results weighted for sampling strategy.

**Serum monoterpenes (ng/mL)**	**Total femur BMD (g/cm** ^**2**^ **)**		
**Ln** α**-pinene**			
Total	718/105988465	−0.027 (0.008)	0.004
Men	354/52250971	−0.021 (0.011)	0.081
Women	364/53737494	−0.035 (0.009)	0.001
Pre-menopausal	109/16281775	−0.040 (0.016)	0.025
Post-menopausal	255/37455719	−0.028 (0.015)	0.074
**Ln- limonene**			
Total	724/106451445	−0.019 (0.007)	0.016
Men	357/52554992	−0.018 (0.010)	0.112
Women	367/53896452	−0.012 (0.009)	0.186
Pre-menopausal	110/16351156	−0.022 (0.024)	0.378
Post-menopausal	257/37545296	−0.002 (0.008)	0.783

Adjusted for model 2.

The associations between monoterpenes and FRAX^®^ scores are presented in Table 6. No correlations were observed among all three monoterpenes and the FRAX^®^ scores. Table 7 presents a summary of the relationships between the history of bone fractures and monoterpenes as observed in the logistic regression models. All three monoterpene levels were associated with an increased incidence of spine fractures (Ln α-pinene: OR = 1.929, 95% CI = 1.270–2.930, *P* = 0.004; Ln β-pinene: OR = 1.970, 95% CI = 1.285–3.020, *P* = 0.004; Ln limonene: OR = 2.613, 95% CI = 1.433–4.768, *P* = 0.004). Additionally, Ln β-pinene was associated with a lower risk of other fractures (OR = 0.579, 95% CI = 0.386–0.868, *P* = 0.012).

**Table 6:** Linear regression coefficients (standard error) for differences in FRAX^®^ scores (hip fracture and major osteoporotic fracture score) relative to one-unit increase in natural log transformed serum monoterpenes in adults 40 years and older, with results weighted for sampling strategy.

	**Ln** α**-pinene (ng/mL)**		**Ln** β**-pinene (ng/mL)**		**Ln- limonene (ng/mL)**	
	β **coeff**	***P*** **value**	β **coeff**	***P*** **value**	β **coeff**	***P*** **value**
**No self-reported fracture after age 20 and no vertebral fracture measured by DXA**						
10-year hip fracture risk score	0.066 (0.151)	0.671	0.126 (0.170)	0.471	0.188 (0.200)	0.361
10-year major osteoporotic fracture risk score	0.018 (0.370)	0.961	0.083 (0.383)	0.832	0.038 (0.476)	0.937
**Previous self-reported fracture after age 20 or vertebral fracture measured by DXA**						
10-year hip fracture risk score	−0.086 (0.403)	0.834	−0.801 (0.373)	0.050	−0.482 (0.297)	0.127
10-year major osteoporotic fracture risk score	−0.296 (0.849)	0.732	−2.204 (1.044)	0.053	−2.439 (1.018)	0.031

**Table 7:** Associations between history of bone fractures and unit increase in natural log-transformed serum monoterpenes in logistic regression models, with results weighted for sampling strategy.

	**Ln** α**-pinene (ng/mL)**		**Ln** β**-pinene (ng/mL)**		**Ln- limonene (ng/mL)**	
	**OR (95% CI)**	***P*** **value**	**OR (95% CI)**	***P*** **value**	**OR (95% CI)**	***P*** **value**
All types of fracture	0.908 (0.675–1.223)	0.501	0.661 (0.446–0.980)	0.040	1.005 (0.688–1.468)	0.978
Spine fracture	1.929 (1.270–2.930)	0.004	1.970 (1.285–3.020)	0.004	2.613 (1.433–4.768)	0.004
Hip fracture	1.364 (0.509–3.660)	0.512	0.546 (0.118–2.513)	0.411	0.461 (0.079–2.680)	0.363
Wrist fracture	0.953 (0.462–1.966)	0.889	0.819 (0.452–1.486)	0.487	0.642 (0.296–1.396)	0.243
Other fracture	0.768 (0.564–1.047)	0.090	0.579 (0.386–0.868)	0.012	0.893 (0.565–1.409)	0.604

Adjusted for model 2.

OR, odds ratio.

## 4 Discussion

Our study, conducted on a on a representative sample of adults in the United States aged 40 and above, unveiled a notable prevalence of serum monoterpenes. Elevated levels of α-pinene and limonene were found to be associated with decreased total proximal femur BMD. Moreover, our results indicate a possible synergistic impact of α-pinene and limonene on total proximal femur BMD. Additionally, all three monoterpenes were associated with a higher occurrence of previous spine fractures, while β-pinene showed a positive correlation with other types of fractures. Although none of monoterpene levels attained statistical significance concerning FRAX^®^ scores, this study presents initial evidence suggesting a potential connection between monoterpenes exposure and bone health in the population of Americans aged 40 and above. Furthermore, we employed three indicators to evaluate bone health, and our analysis benefited from meticulous control of numerous potential variables within the comprehensive NHANES database.

α-Pinene and β-pinene are cyclic monoterpenes that are prominent constituents of emissions from conifer trees (31). Limonene is a monoterpene consisting of a single ring, naturally occurring in two enantiomeric forms. L-limonene occurs naturally in the pine trees, while D-limonene is a byproduct of citrus juice extraction and is also found in caraway oil (5). Increased concentrations of monoterpene compounds in outdoor air can arise from natural biogenic sources as well as human-induced anthropogenic activities (16, 17). In addition to outdoor air, monoterpene exposure occurs indoors as well, as they are commonly found in domestic cleaners and room fragrances (32). Apart from respiratory exposure, individuals can also come into contact with monoterpene through oral intake and skin contact, as they are utilized in essential oils, fragrances, food flavorings, and additives (33). The current study exclusively examines internal exposure to monoterpene, uncovering a high detection rate and indicating inevitable exposure in everyday life.

There have been several in experimental studies explored the effect of monoterpene exposure on bone cells. The addition of α-pinene to osteogenic medium resulted in heightened expression of osteogenic markers, indicating improved differentiation and mineralization of osteoblasts. Furthermore, α-pinene mitigates the inhibitory effects of tumor necrosis factor α on osteoblast differentiation (13). In mouse preosteoblasts, limonene notably boosted cell proliferation, enhanced bone extracellular matrix synthesis and mineral deposition (14). In rats, cis-verbenol, a metabolite of α-pinene, contrasts with the parent compound by inhibiting osteoclast activity (15).

On the other hand, it's crucial to recognize the potential negative impacts of monoterpenes. These compounds can produce secondary organic aerosols either via oxidation processes or through interaction with ozone (16–18). An *in vitro* study revealed that exposure to α-pinene secondary organic aerosol reduced proliferation in a lung cell line. This exposure also resulted in heightened oxidative stress, likely attributable to organic hydroperoxides present in the secondary organic aerosol (19). Moreover, within human metabolism, α-pinene undergoes conversion into the reactive metabolite α-pinene oxide, alongside other metabolites, thereby potentially increasing toxicity and oxidative stress (20–22). While α-pinene alone may not trigger adverse reactions, its metabolites produced both externally and internally could potentially induce heightened oxidative stress, which is a significant factor in the decline of BMD (11, 12). Our study offers the initial epidemiological evidence indicating that serum levels of α-pinene and limonene are inversely correlated with proximal femur BMD. Further research is essential to enhance our understanding of precise mechanism associated with monoterpenes.

We found that individuals with α-pinene and limonene above the 50th percentile had a significantly higher mean total proximal femur BMD compared to those at or below the 50th percentile. These results indicate a possible synergistic effect between these two monoterpenes concerning BMD. In a previous *in vitro* study, the concurrent administration of α-pinene and β-pinene with paclitaxel resulted in a synergistic apoptosis effect against lung cancer cell line (34). In our earlier investigation, also drawing on NHANES 2013-2014 data, individuals whose levels of all three monoterpenes exceeded the 50th percentile displayed markedly elevated lipid profiles in contrast to those fell below the 50th percentile (7). In this study, the detected synergy between α-pinene and limonene regarding total proximal femur BMD may stem from their collective biological functions. Collaboratively, they may exert a more substantial impact on BMD than when acting individually.

Our study found elevated levels of α-pinene and limonene were found to be associated with decreased proximal femur BMD, but not lumbar spine. The difference may stem from several factors. Firstly, the proximal femur experiences greater mechanical loading due to weight-bearing activities, making it more vulnerable to environmental influences like monoterpene exposure compared to the lumbar spine (35). Secondly, bone remodeling dynamics differ between cortical and trabecular bone, which are predominant in the proximal femur and lumbar spine, respectively (36). These differences in bone structure and turnover rates could lead to variations in how monoterpene exposure impacts BMD in these regions. Thirdly, the sensitivity of bone cells and signaling pathways to monoterpene exposure may vary locally within different skeletal sites. For example, the trabecular section of long bones serves as a readily accessible store of calcium for physiological purposes, in contrast to other skeletal components (37). If monoterpene exposure affects osteoblast activity differently in the proximal femur vs. the lumbar spine, it could result in divergent effects on BMD.

One intriguing discovery from the current analyses is the pronounced association between α-pinene concentrations and decreased BMD, particularly notable in women, especially those in the premenopausal stage. Various potential explanations could account for this observation. Firstly, α-pinene has been shown to inhibit steroidogenic enzymes, which is responsible for converting testosterone to estrogen (38). Given that premenopausal women generally have elevated estrogen levels, any interference by α-pinene with estrogen metabolism may lead to more noticeable impacts on BMD compared to men or postmenopausal women. Secondly, inherent disparities in bone metabolism between genders, such as the lower peak bone mass and accelerated bone loss observed in women (39), could potentially heighten their susceptibility to the effects of α-pinene. Finally, while the effect of α-pinene on BMD may be weaker than estrogen deficiency, the trend observed in menopausal women within this cohort may not have achieved statistical significance due to sample limitations. Thus, further comprehensive research is warranted to elucidate the underlying mechanisms driving these associations and their implications for women's bone health, particularly during the premenopausal stage.

Our findings revealed no association between monoterpene exposure and FRAX^®^ score. In bisphosphonate clinical trials, variations in clinical outcomes were reflected by BMD differences of ≥ 5% in menopausal women with osteoporosis (40). In this current research, the disparity in proximal femur BMD between the lowest and highest quartiles of α-pinene exposure was only 5 % in the study population. This impact of α-pinene exposure on femur BMD might not be substantial enough to significantly affect future clinical outcomes. Furthermore, we observed that higher serum monoterpene concentrations were associated with an increased risk of previous spine fractures, whereas β-pinene was linked to a lower risk of other fracture types. It's challenging to attribute an old fracture to a solitary measurement of monoterpene exposure. It's plausible that the recorded status of monoterpene exposure status is closely tied to past exposure, thereby correlating with historical fractures. Nevertheless, given the sequential association between monoterpene exposure and fractures, no definitive inferences can be drawn. Further investigation is needed to clarify these relationships.

Our study faces several limitations. Firstly, our study was confined to serum monoterpene levels and DXA examinations in adults from NHANES 2013–2014, potentially restricting the relevance of our findings to diverse age cohorts and regions. Additionally, the retrospective cross-sectional nature of our study prevents the establishment of causal connections. Furthermore, our comprehensive set of covariates addresses the most significant influences on both monoterpene levels and BMD. However, residual confounding may still exist due to unmeasured dietary and lifestyle factors. Moreover, we evaluate the association between monoterpenes and bone health using data from NHANES 2013-2014. The rationale for choosing this specific period is that monoterpene data is only available in this cycle. Despite the data being from ten years ago, monoterpenes remain substances that people encounter daily, and our findings provide the first epidemiological evidence of their impact on bone health. Lastly, it is important to recognize that monoterpenes are rapidly metabolized into oxidized forms, which may have different effects on bone health. However, the NHANES database does not include data on these metabolites, limiting our analysis.

## 5 Conclusion

Our retrospective cross-sectional analysis on NHANES 2013-2014 data revealed a significant detection rate of serum monoterpenes, with elevated levels of α-pinene and limonene correlating with decreased total proximal femur BMD. Moreover, our results indicate a potential combined impact of α-pinene and limonene on total proximal femur BMD, suggesting a synergistic effect. In addition, all three monoterpenes are associated with a higher incidence of previous spine fractures. Although certain monoterpene compounds may possess advantageous characteristics, others can be harmful. Further longitudinal investigation is required to determine the clinical importance and causation of our findings.

## Data availability statement

Publicly available datasets were analyzed in this study. This data can be found here: https://www.cdc.gov/nchs/nhanes/index.htm.

## Ethics statement

Ethical approval was not required for the studies involving humans because NHANES is an analysis from a publicly available database. The studies were conducted in accordance with the local legislation and institutional requirements. The participants provided their written informed consent to participate in this study.

## Author contributions

Y-WF: Formal analysis, Methodology, Validation, Writing – original draft. C-KW: Conceptualization, Data curation, Methodology, Resources, Supervision, Writing – original draft. C-YL: Data curation, Methodology, Project administration, Visualization, Writing – review & editing.
