# Supplementary material for: The relationship between serum monoterpene levels and bone health: a retrospective cross-sectional analysis from the National Health and Nutrition Examination Survey (NHANES) data
Source: Front Public Health. 2024 Aug 7;12:1436415. doi: 10.3389/fpubh.2024.1436415 (PMC11335497; doi:10.3389/fpubh.2024.1436415)
Supplement: Supplementary file 1 [file Table_1.DOCX]

**The Relationship Between Serum Monoterpene Levels and Bone Health: Findings from a U.S. Nationally Representative Survey**

**Online Supplementary Material**

**2. Materials and methods**

***2.5. Other covariates.***

According to the statements on the NHANES website, data were collected at all study sites by trained personnel using standardized procedures. Sociodemographic information, such as age, sex, and race/ethnicity, was collected during the household interview. Body mass index (BMI) was calculated as weight in kilograms divided by height in meters squared. The smoking status was categorized as active smoker, secondhand smoker and non-smoker based on a smoking questionnaire (1). Alcohol consumption was determined by the questionnaire (“Had at least 12 alcohol drinks/1 yr?”) and was dichotomized. Menopause was assessed by women choosing menopause/change of life as an answer (“What is the reason that you have not had a period in the past 12 months?”) and choosing yes as an answer (Had both ovaries removed?) in the questionnaire. However, some women choosing hysterectomy as an answer (“What is the reason that you have not had a period in the past 12 months?”) and others missing from reproductive health questionnaire in the study. In these women, menopause was defined if the serum estradiol level was < 30 pg/mL (2). Serum 25(OH) D levels (25OHD2+25OHD3) were measured by liquid chromatography-tandem mass spectrometry and divided into three groups (<50, 50–75, >75 nmol/L). Total calcium intake was counted by the USDA’s Food and Nutrient Database for Dietary Studies 2013–2014 and was averaged from the two-day intake amount in this study. Ever treated for osteoporosis was determined from questionnaire of prescription medications and the questionnaire (“Have you ever been told by a doctor or other health care professional to take a prescribed medicine for osteoporosis?”), as was ever taking prednisone or cortisone was determined from questionnaire of prescription medications and the questionnaire (“Have you ever taken any prednisone or cortisone pills nearly every day for a month or longer?”). Using female hormones after menopause was determined from questionnaire of prescription medications and the questionnaire (“Ever used female hormones such as estrogen and progesterone? Please include any forms of female hormones, such as pills, cream, patch, and injectables, but do not include birth control methods or use for infertility”).

Physical Activity was assessed in each NHANES participant by a physical activity questionnaire which was based on the Global Physical Activity Questionnaire (GPAQ) (3). Vigorous work activity was determined from the questionnaire (“Does your work involve vigorous-intensity activity that causes large increases in breathing or heart rate like carrying or lifting heavy loads, digging or construction work for at least 10 minutes continuously?). Moderate work-related activity was determined from the questionnaire (“Does your work involve moderate-intensity activity that causes small increases in breathing or heart rate such as brisk walking or carrying light loads for at least 10 minutes continuously?). Walking or bicycling for transportation was determined from the questionnaire (“In a typical week do you walk or use a bicycle for at least 10 minutes continuously to get to and from places?). Vigorous leisure-time physical activity was determined from the questionnaire (In a typical week do you do any vigorous-intensity sports, fitness, or recreational activities that cause large increases in breathing or heart rate like running or basketball for at least 10 minutes continuously?). Moderate recreational activity was determined from the questionnaire (In a typical week do you do any moderate-intensity sports, fitness, or recreational activities that cause a small increase in breathing or heart rate such as brisk walking, bicycling, swimming, or volleyball for at least 10 minutes continuously?)

The suggested metabolic equivalent (MET) scores for vigorous work-related physical activity, moderate work-related physical activity, walking or bicycling for transportation, vigorous leisure-time physical activity, and moderate leisure-time physical activity were 8.0,4.0, 4.0, 8.0, and 4.0, respectively (3). The average number of hours per week spent in each activity was multiplied by the suggested MET scores to get an estimate of MET-hours per week.

In NHANES 2013-2014 database, past history of fracture was coded in “Osteoporosis” questionnaire (4). The target sample for this section is participants aged 40 and over. history of hip, wrist, or spine fracture was determined from the questionnaire (“Has a doctor ever told you that you had a broken or fractured hip, wrist, or spine?”). History of other fractures from the questionnaire (“Has a doctor ever told you that you had broken or fractured any other bone after 20 years of age?”). All types of fracture were determined by the sum of hip, wrist, spine fractures, and other fractures.

**References:**

1. National Health and Nutrition Examination Survey (Nhanes): Smoking Center of Disease Control and Prevention(2016) [cited 2020 May 28]. Available from: <http://wwwn.cdc.gov/nchs/nhanes/search/DataPage.aspx?Component=Questionnaire&CycleBeginYear=2013>.

2. Lee JS, Ettinger B, Stanczyk FZ, Vittinghoff E, Hanes V, Cauley JA, et al. Comparison of Methods to Measure Low Serum Estradiol Levels in Postmenopausal Women. *The Journal of clinical endocrinology and metabolism* (2006) 91(10):3791-7. Epub 2006/08/03. doi: 10.1210/jc.2005-2378.

3. Wang J, Wu Y, Ning F, Zhang C, Zhang D. The Association between Leisure-Time Physical Activity and Risk of Undetected Prediabetes. *Journal of diabetes research* (2017) 2017:4845108. Epub 2017/04/04. doi: 10.1155/2017/4845108.

4. National Health and Nutrition Examination Survey: Osteoprosis (2013-2014) [cited 2020 July 30]. Available from: <https://wwwn.cdc.gov/Nchs/Nhanes/2013-2014/OSQ_H.htm#OSD030aa>.
